# The Universally Conserved Unconventional G Protein YchF Is Critical for Growth and Stress Response

**DOI:** 10.3390/life13041058

**Published:** 2023-04-20

**Authors:** Zhaoheng Lin, Rongfang Li, Zhiwei Han, Yi Liu, Liyang Gao, Suchang Huang, Ying Miao, Rui Miao

**Affiliations:** Fujian Provincial Key Laboratory of Plant Functional Biology, College of Life Sciences, Fujian Agriculture and Forestry University, Fuzhou 350002, China

**Keywords:** YchF, growth, stress response, P-loop NTPase, ribosome, proteosome, protein translation, protein degradation

## Abstract

The ancient guanine nucleotide-binding (G) proteins are a group of critical regulatory and signal transduction proteins, widely involved in diverse cellular processes of all kingdoms of life. YchF is a kind of universally conserved novel unconventional G protein that appears to be crucial for growth and stress response in eukaryotes and bacteria. YchF is able to bind and hydrolyze both adenine nucleoside triphosphate (ATP) and guanosine nucleoside triphosphate (GTP), unlike other members of the P-loop GTPases. Hence, it can transduce signals and mediate multiple biological functions by using either ATP or GTP. YchF is not only a nucleotide-dependent translational factor associated with the ribosomal particles and proteasomal subunits, potentially bridging protein biosynthesis and degradation, but also sensitive to reactive oxygen species (ROS), probably recruiting many partner proteins in response to environmental stress. In this review, we summarize the latest insights into how YchF is associated with protein translation and ubiquitin-dependent protein degradation to regulate growth and maintain proteostasis under stress conditions.

## 1. Introduction

The ancient guanine nucleotide-binding (G) proteins, namely phosphate-binding-loop guanosine triphosphatases (P-loop GTPases), play crucial roles in protein synthesis and cellular signaling transduction among all kingdoms of life [1]. G proteins possess an active guanosine triphosphate (GTP) bound state and inactive guanosine diphosphate (GDP) bound state in a cyclic manner through loading GTP and hydrolyzing GTP to GDP [2,3]. G proteins receive upstream environmental signaling and transduce to downstream effectors. G protein molecular switches between “on” and “off” are coordinated by three G-protein-regulator families, including GTPase-activating proteins (GAPs), guanine nucleotide exchange factors (GEFs), and guanosine nucleotide dissociation inhibitors (GDIs). Nucleotide hydrolysis is accelerated by GAPs with the results of signal termination, while GEFs relieve GDP and replace GTP with G proteins, thereby activating the G proteins and turning on the signal transduction [1,4,5]. In contrast to GEFs, GDIs prevent the exchange of GTP with GDP and maintain G proteins in an inactive GDP-bound state, but the inhibition is revertible by the GEFs depending on the environmental stimuli [2,6,7].

On the basis of sequence and structural features, G proteins can be divided into two large distinct superclasses: The translation Factors (TRAFAC) superclass and Signal Recognition GTPases and the MinD and BioD (SIMIBI) superclass [8]. Secondly, signal-transducing G proteins comprise heterotrimeric G proteins, small G proteins, and many unconventional G proteins [4,9]. First, heterotrimeric G proteins are composed of G protein α subunits (Gα), Gβ and Gγ subunits. The GTPase (G) domain of heterotrimeric G protein is in the Gα subunit. The seven-transmembrane-spanning (7TM) G protein-coupled receptors (GPCR) work as GEFs to catalyze the exchange of GDP for GTP on the Gα subunit, while regulator of G-protein signaling (RGS) stimulates Gα subunit GTPase activity as GAPs [10,11]. In the presence of GDP, the GDP-bound Gα subunit integrates with the Gβγ heterodimer as a ternary complex intracellularly anchoring to GPCR at the plasma membrane. In the presence of GTP, however, the GTP-bound Gα subunit goes through a conformational change that allows heterotrimeric G protein dissociation into the Gα subunit and Gβγ heterodimer. Then, the Gα subunit and Gβγ heterodimer couple to their own effectors for signaling transduction [12]. Secondly, small G proteins hold a ~170 amino acid residue core G domain along with extra N-terminal and C-terminal extensions and can be divided into five families, such as Ras (Rat sarcoma), Rho (Ras homology), Arf (ADP-ribosylation factor), Rab (Ras-like in the brain), and Ran (Ras-like nuclear) [13]. Finally, heterotrimeric G proteins and small G proteins belong to the extended Ras-like family in the TRAFAC superclass, whereas the unique, unconventional G protein YchF subfamily is a part of the Obg family in TRAFAC superclass of G proteins [8,14].

## 2. Structure of G Domain among G Proteins

### 2.1. Structural Characterization of G Domain of G Proteins

**Figure 1:**
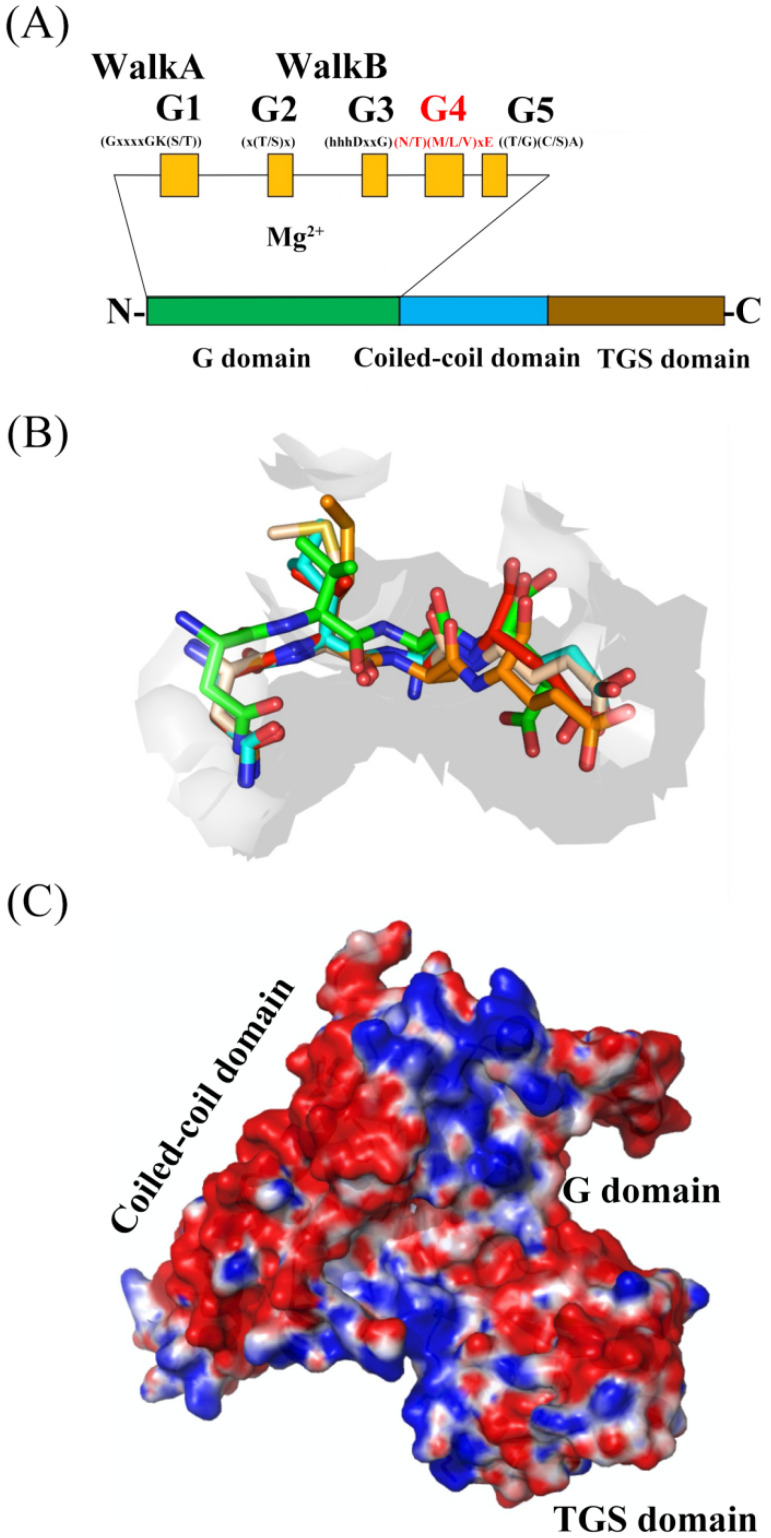
Structural characterization of the evolutionarily conserved unconventional G protein YchF. (**A**) Schematic representation of the structure of universally conserved unconventional G protein YchF. (**B**) Structural alignment of the nontypical G4 motif (N/T)(M/L/V)xE in the YchF subfamily (*E. coli* YchF is green, hOLA1 is cyan, *S. pombe* YchF is yellow, OsYchF1 is brown, *T. thermophilus* YchF is red). The amino acid residues are shown as sticks. (**C**) Electron density surface of the apo-structure of OsYchF1 (Protein Data Bank (PDB) code: 5EE0). Negatively charged amino acid residues are red, and positively charged amino acid residues are blue.

All G proteins utilize the G domain to bind and hydrolyze nucleotides, which contains five structurally conserved motifs (G boxes): G1 motif (G1 box) adopting the sequence pattern GxxxxGK(S/T), G2 motif (G2 box) adopting the sequence pattern x(T/S)x, G3 motif (G3 box) adopting the sequence pattern hhhDxxG, G4 motif (G4 box) adopting the sequence pattern (N/T)KxD and G5 motif (G5 box) adopting the sequence pattern (T/G)(C/S)A [5] (Figure 1A). The so-called P-loop or walk A motif is the G1 box that binds to α- and β-phosphate of nucleotides. The walk B motif consists of a G2 box and a G3 box that anchor to the terminal γ-phosphate of nucleotide. The G3 box has a conserved aspartic acid (Asp/D) residue in contact with the co-factor magnesium (Mg^2+^), which is crucial for nucleotide binding and hydrolysis (Figure 1A). In addition, the walk B motif overlapping with the switch I and switch II regions undergo a conformational change accompanied by nucleotide hydrolysis, which governs effector binding. The G4 box determines the guanosine or adenosine signature, and the G5 box supports specific recognition.

### 2.2. Structural Characterization of G Domain of YchF

The G domain of universally conserved unconventional G protein YchF maintains five fingerprint motifs as other G proteins. The G1, G2, G3, and G5 boxes are invariant with other G proteins, but the G4 box in the YchF subfamily shows a nontypical (N/T)(M/L/V)xE amino acid sequence instead of (N/T)KxD (Figure 1A,B; Table 1). Thus, the members in the YchF subfamily are capable of binding and hydrolyzing both adenine nucleoside triphosphate (ATP) and guanosine nucleoside triphosphate (GTP) [9].

### 2.3. Structural Comparison of G Domains among Selected YchF, Small G Protein, and Heterotrimeric G Protein α-Subunit

**Figure 2:**
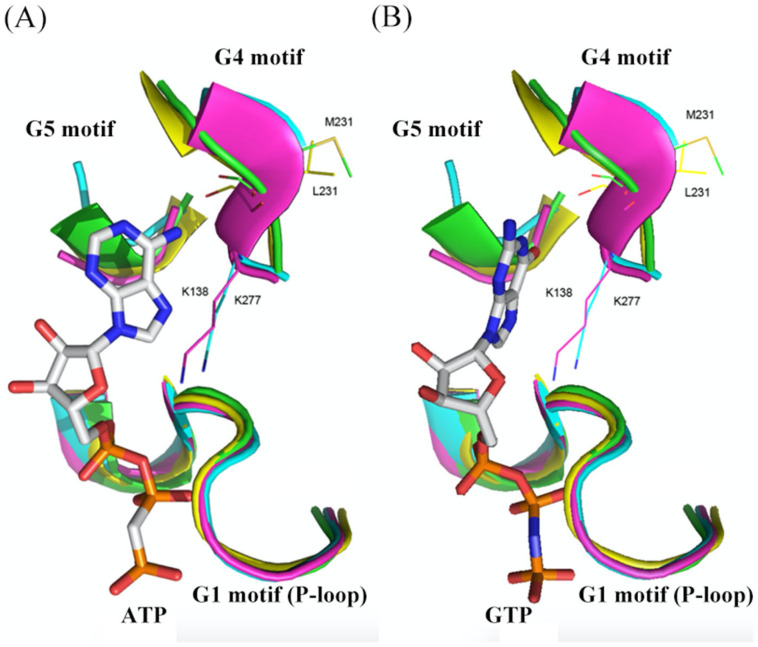
Structural alignments of OsYchF1 hOLA1 *H. sapiens* Ras-related G protein C and *R. norvegicus* heterotrimeric G protein α-subunit nucleotide-binding sites in the complex with nucleotides. (**A**) Structural alignments of OsYchF1 (PDB code: 5EE3), hOLA1 (PDB code: 2OHF), human Ras-related G protein C (HsRas C) (PDB code: 3LLU), and *R. norvegicus* heterotrimeric G protein α-subunit (RnHetero) (PDB code: 1SVS) nucleotide-binding site in the complex with the ATP non-hydrolyzed homolog AMPPNP. (**B**) Structural alignments of OsYchF1 (PDB code: 5EE9), hOLA1, HsRas C, and RnHetero nucleotide-binding site in the complex with the GTP non-hydrolyzed homolog GppNHp. AMPPNP, GppNHp, M-231, L-231, K-138, and K-277 are shown as sticks. The G1 motif (P-loop), G4 motif, and G5 motif are shown as cartoons (OsYchF1 is green, hOLA1 is yellow, human Ras-related G protein C is cyan, and *R. norvegicus* heterotrimeric G protein α-subunit is pink).

Herein, a heterotrimeric G protein α-subunit in the rat (*Rattus norvegicus*) and a human (*Homo Sapien*) small G protein Ras-related G protein C was chosen to compare with OsYchF1, a rice (*Oryza sativa*) ortholog of YchF in plants. In contrast to *R. norvegicus* heterotrimeric G protein α-subunit and human Ras-related G protein C, the novel G4 motif and G5 motif of OsYchF1 support either ATP or GTP binding in the nucleotide-binding site of OsYchF1 (Figure 1B and Figure 2A). According to the crystal structure of OsYchF1 in the presence of the ATP non-hydrolyzed homolog AMPPNP (Protein Data Bank (PDB) code: 5EE3), the backbone carboxyl group of methionine (M231) in the G4 motif of OsYchF1 forms a hydrogen bond with the adenine 6-amino group of AMPPNP (Figure 2A) [9,20]. This allows for the non-hydrolytic AMPPNP to be able to fit into the OsYchF1 nucleotide-binding site (Figure 2A). The structural alignments of OsYchF1 with *R. norvegicus* heterotrimeric G protein α-subunit (PDB code: 1SVS) and human Ras-related G protein C (PDB code: 3LLU) revealed that the side chain of asparagine (Asn) in the G4 motif of OsYchF1 could not turn back and interact with the 2-amino group of guanosine, unlike the other two proteins. However, the crystal structure of OsYchF1 in the presence of GppNHp (PDB code: 5EE9), a non-hydrolyzed homolog of GTP, showed that the G5 motif of OsYchF1 can form a hydrogen bond with the guanosine base group of GppNHp. This finding partially explains why OsYchF1 is capable of binding to GTP as well (Figure 2B) [9,20]. Moreover, the G1 motif (P-loop) is highly conserved and consistent among OsYchF1, *R. norvegicus* heterotrimeric G protein α-subunit, and human Ras-related G protein C (Figure 2A,B). The G1 motif of OsYchF1 interacts with the triphosphate of nucleotides, resembling the G1 motif of *R. norvegicus* heterotrimeric G protein α-subunit and human Ras-related Protein C (Figure 2A,B). In the OsYchF1 G4 motif mutant, however, methionine (Met) and glutamine (Glu) were replaced by lysine (Lys) and aspartic acid (Asp), respectively. With this change in amino acids, OsYchF1 obtained GTP priority again, indicating that the OsYchF1 G4 motif indeed determines ATP or GTP recognition [9,20].

## 3. YchF Is Critical for Growth and Stress Response

### 3.1. YchF Works as a Conserved Negative Regulator in Response to Oxidative Stress

YchF is a universally conserved unconventional G-proteins in most organisms except archaea and consists of an N-terminal core G domain, inserted large coiled-coil domain, and C-terminal TGS (ThrRS, GTPase, and SpoT) domain potentially favoring the RNA binding and ubiquitin-dependent protein degradation (Figure 1A,C). Thus far, the available results suggest that YchF is probably a nucleotide-dependent translational factor associated with the ribosome and proteasome and likely links with other partner proteins as a unique negative regulator of the oxidative stress response (Table 2).

hOLA1 (human Obg-like ATPase1) (~45 kDa) is a human ortholog of YchF that is expressed in the cytoplasm [30,34]. The *hOLA1* overexpression cells showed increased sensitivity to oxidant-induced cytotoxicity. Conversely, *hOLA1*-knockdown cells conferred tolerance to oxidizing agents, such as tert-butyl hydroperoxide (tBH) and diamide, and *hOLA1*-knockdown cells demonstrated reduced cellular reactive oxygen species (ROS) production [34].

**Figure 3:**
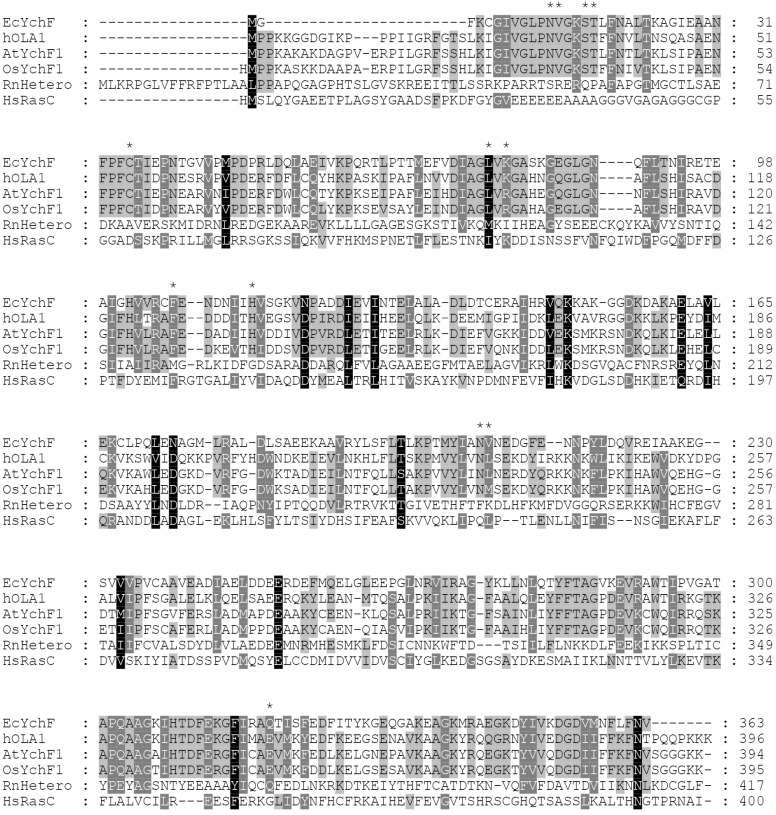
Sequence alignments of *E. coli* YchF (EcYchF), hOLA1, OsYchF1, AtYchF1, *H. sapiens* Ras-related G protein C and *R. norvegicus* heterotrimeric G protein α-subunit using software Jalview version 1.6 (https://www.jalview.org). *E. coli* YchF NCBI Protein code is VWQ02248.1, hOLA1 NCBI Protein code is NP_037473.3, OsYchF1 NCBI Protein code is BAD03576.1, AtYchF1 NCBI Protein code is Q9SA73.1, human Ras-related G protein C (HsRas C) NCBI Protein code is NP_071440.1, and *R. norvegicus* heterotrimeric G protein α-subunit (RnHetero) NCBI Protein code is XP_010846404.1. The conserved amino acid residues are marked in the dark, and the more conserved amino acid residues are much darker, and the specific amino acid residues in Table 1 were marked with *.

*Escherichia coli YchF* expression is growth phase-dependent and down-regulated under oxidative stress conditions [30]. *E. coli YchF* overexpression enhanced cellular sensitivity to H_2_O_2_-induced oxidative stress, while the *E. coli YchF* deletion strain displayed increased resistance against H_2_O_2_ and diamide [18]. Although *E. coli* YchF physically interacts with the *E. coli* catalase KatG and *E. coli YchF* overexpression inhibits KatG enzyme activity in vivo, there is no effect on KatG enzyme activity in the presence of the purified *E. coli* YchF in vitro, suggesting that the reduced catalase activity should be an indirect effect in vivo (Figure 3; Table 2) [18].

Moreover, *E. coli* YchF functions as a redox-regulated monomer-dimer equilibrium through a conserved cysteine residue 35 within the *E. coli* YchF nucleotide-binding site (Table 1) [16]. The *E. coli* YchF dimer shows a low ATPase activity, but the *E. coli* YchF monomer displays significantly increased *E. coli* YchF ATPase activity (Figure 3; Table 2) [35]. Thioredoxin 1 (TrxA) maintains the redox balance in vivo and directly interacts with the G domain and coiled-coil domain of *E. coli* YchF to dissociate *E. coli* YchF dimer. Interestingly, wild-type *E. coli* cells effectively outcompete the *E. coli* YchF deletion strain, indicating that *E. coli* YchF might influence *E. coli* cell growth, but the mechanism is unclear [30].

### 3.2. YchF Is Crucial for Environmental Stress Response

A fatal marine bacterium *Vibrio vulnificus,* ortholog of YchF, elicits macrophage cytotoxicity. It shows a significant negative effect of macrophage cytotoxicity on iron-overloaded mice through the *rtxA1* pathway that stimulates cytotoxicity to macrophages [31,36]. The *V. vulnificus YchF* deletion strain displayed retarded growth and reduced transcription level of the *rtxA1* gene [31,36]. In addition, *Propionibacterium acidipropionici* ortholog of YchF is crucial for the regulation of propionic acid tolerance [31,36].

The expression of *hOLA1*, namely *DNA damage-regulated overexpressed in cancer 45* (*DOC45*), was strongly down-regulate by DNAdamage-inducing agents, such as etoposide, doxorubicin (adriamycin), and ionizing and UV radiation, but not endoplasmic reticulumstress-inducing agents [20]. Compared with normal human cells, *hOLA1* expression is notably upregulated in established colon cancer cells at both mRNA and protein levels [20]. *hOLA1*-knockdown human colon cancer cells show a negative impact on cell proliferation and hypersensitivity to Adriamycin-induced cell death [20,34,37].

### 3.3. YchF Bridges Protein Biosynthesis and Degradation

YchF anchors to ribosomes and polysomes, suggesting that YchF is involved in protein biosynthesis (Table 2) [17,24]. The ribosome is likely in contact with the N-terminal G domain of YchF [34]. Consistently, the 70 S ribosomal subunit is able to enhance *E. coli* YchF ATPase activity, although *E. coli* YchF hardly influences the assembly and steady-state amounts of ribosomes [38]. *E. coli* YchF preferentially binds to the translation initiation factor 3 (IF3) and several ribosomal proteins at the surface of the 30 S ribosomal particle, while the interaction of *E. coli* YchF with 50 S ribosomal particle seems probably only transient [17,24].

The percentage of leaderless mRNAs is only 0.7% in *E. coli* BW25113 under normal growth conditions, but the ribonuclease MazF generates leaderless mRNAs by cleaving off the Shine-Dalgarno (SD) sequence close to the start-codon upon environmental stress [39,40]. The MazF-generated leaderless mRNA modulation is necessary for bacterial survival under environmental stress [41]. Compared with wild-type *E. coli* cells, the *E. coli YchF* deletion strain showed increased resistance against MazF-generated leaderless mRNAs [30]. In other words, *E. coli* YchF suppresses the translation of MazF-processed mRNAs upon stress conditions and declines the resistance towards the endoribonuclease [30] (Figure 3; Table 2). In addition, the *E. coli YchF* deletion strain demonstrated increased resistance to hydroxyurea (HU), a ribonucleotide reductase inhibitor, and fusidic acid, an elongation factor G (EF-G) inhibitor [42,43].

A tandem-affinity purification and mass spectrometry (TAP-MS) approach shows the interaction between yeast *Saccharomyces cerevisiae* ortholog of YchF (YBR025C) and eukaryotic translation elongation factor 1 (eEF1), committed with protein translation (Table 2) [28,29]. Additionally, hOLA1 interacts with eukaryotic initiation factor 2 (eIF2) mediates ribosomal recruitment of the initiator methionyl-tRNA (tRNAi) and interferes with the eIF2-mediated formation of a ternary complex with GTP and tRNAi [26] (Figure 3; Table 2).

In the protozoan *Trypanosoma cruzi*, the ortholog of YchF (~44.3 kDa) is associated with not only ribosomal particles and polysomes but also proteasomal subunits allowing protein degradation by the ubiquitin-proteasome pathway (Table 2) [26]. Immunoprecipitation assays exhibited that *T. cruzi* YchF co-sediments with the non-ATPase subunit RPN10 of the *T. cruzi* proteasome, which might mediate damaged protein degradation during protein biosynthesis under stress conditions [26]. An integrated mass spectrometry-based proteomic approach also indicated that the 26S proteasome links with *S. cerevisiae* YchF (YBR025C) in yeast [27]. Due to the structural similarity of the TGS domain to ubiquitin-like proteins, the C-terminal TGS domain of YchF is a potential candidate for the interaction between YchF and the subunits of the proteasome [44]. Noticeably, the absence of *T. cruzi YchF* restrains the cellular growth of *T. brucei* as well as the procyclic forms of the parasite [26]. As hOLA1 and *E. coli* YchF, *T. cruzi* YchF also bind and hydrolyzes ATP more efficiently than GTP [14,17].

### 3.4. YchF Is a Key Molecule in Maintaining Proteostasis

The well-known heat-shock response is a major strategy towards environmental stimuli by the rapid biosynthesis of the molecular chaperone heat-shock proteins [32]. Heat-shock proteins are essential for maintaining intracellular homeostasis by assisting in the damaged proteins [32]. Heat-Shock Protein 70 (HSP70) plays a key role in multiple primary human cancers, and high expression of HSP70 is related to poor tumor progression [33,37]. hOLA1 interacts with the C-terminal variable domain of HSP70 to prevent contact with the C-terminus of Hsp70-binding protein (CHIP), an E3 ubiquitin ligase for HSP70, thereby inhibiting HSP70 from the CHIP-mediated ubiquitination. Thus, hOLA1 stabilizes HSP70 to improve survival under stress conditions [32]. Additionally, Hsp70 is also a molecular chaperone for mitochondrial superoxide dismutase 2 (SOD2), which is responsible for keeping normal mitochondrial reactive oxygen species (ROS) [33]. hOLA1 directly recruits Hsp70 and SOD2 to hinder them from ubiquitin-dependent protein degradation under stress conditions [33]. In conclusion, YchF controls multiple proteostatic mechanisms in response to environmental stresses.

## 4. OsYchF1/AtYchF1 and Its Activator OsGAP1/AtGAP1 in Plants

In nature, plants are often exposed to various environmental stresses during growth and development, including flooding, drought, salt, cold, insect herbivores, and microbes [45,46]. Plants have to evolve sophisticated mechanisms to guard themselves against these environmental challenges [45,47]. Rice (*O. sativa*) GTPase-activating protein 1 (OsGAP1), a C2 domain-containing protein involved in plant defense response pathway, was originally identified using suppression subtraction hybridization (SSH) of a Xa14 rice cDNA library derived from a rice line harboring the *Xa14* resistance gene against the bacterial pathogen *Xanthomonas oryzae* pv. *oryzae* (*Xoo*) [1]. OsGAP1 was constructed as a bait to capture the prey OsYchF1 by yeast two-hybrid assay. The transgenic *Arabidopsis thaliana* ectopically overexpressing *OsGAP1* showed increased resistance with upregulating expressions of both salicylic acid (SA)-related (*PR1* and *PR2*) and jasmonic acid (JA)-related (*Thi2.1* and *PDF1.2*) defense marker genes on *Pst* DC3000 was dependent on [1]. Furthermore, the resistant effects of *OsGAP1* on *Pst* DC3000 are dependent on the key plant biotic stress response regulator *NONEXPRESSOR OF PATHOGENESIS-RELATED GENES* 1 (*NPR1*) [1]. *OsGAP1* ectopically overexpressed in the *A. thaliana npr1-3* mutant never showed increased resistance towards *Pst* DC3000 [1].

OsYchF1 almost utilizes ATP and GTP equally, unlike protozoan, bacterium, and human YchF orthologs that give priority to ATP over GTP [1]. OsGAP1 significantly enhances OsYchF1 ATPase and GTPase activities and turns OsYchF1 into the inactive GDP or ADP-bound state [1]. Moreover, OsGAP1 might control the subcellular localization of OsYchF1 by recruiting cytosolic OsYchF1 to the intracellular plasma membrane subjected to wounding treatment [1]. OsYchF1 and its activating protein OsGAP1 play opposite roles in response to environmental stimuli (Figure 3; Table 2). On the one hand, the *OsYchF1* overexpressors are sensitive to the bacterial pathogen *Pseudomonas syringae* pv. *tomato* DC3000 (*Pst* DC3000), but the *A. thaliana YchF* (*AtYchF1*) knockout mutant and OsGAP1 overexpressors confer tolerance to the bacterial pathogen in *A. thaliana* [1,48]. On the other hand, the overexpression of *OsYchF1* and *AtYchF1* in transgenic *A. thaliana* results in decreased resistance to high salinity-induced oxidative stress, while the overexpression of *OsGAP1* or *AtGAP1* (*OsGAP1* ortholog in *A. thaliana*) and the *AtYchF1* knockout mutant alleviate salt stress [21].

In order to dissect the interaction of OsYchF1 with OsGAP1, firstly, site-directed mutagenesis identifies three clusters (D23, D28; R117, N119, E123, E124; R141, R143, E146, E149) of OsGAP1 surface amino acid residues that are essential for binding to phospholipids, which play an important role in enhancing defense responses. Additionally, the effects of OsGAP1 on high salinity tolerance are dependent on the interaction between the other two clusters (L5, L8, T58, S60, and Ser-60; K37, K39, K41, R43) of OsGAP1 and OsYchF1 [19,20]. Secondly, a recent study explains that four critical amino acid residues (Lys-325, His-334, Glu-345, and Glu-354) in the OsYchF1 TGS domain are required for the interaction of OsGAP1 with OsYchF1 [19].

Slot blot analysis demonstrates that the OsYchF1 TGS domain interacts with the 26S RNA in rice, suggesting that OsYchF1 is committed to protein biosynthesis as well [1]. Additionally, recent co-crystallization and biochemical data showed that AtYchF1 in the complex with ppGpp inhibits the interaction of AtYchF1 with other molecules, including ATP, GTP, and 26S rRNA [23]. The available data indicate that ppGpp works as an alarmone in response to environmental stimuli, and the concentration of ppGpp in the cytoplasm can increase to the millimolar level upon stress conditions [3,23,49]. Most importantly, the accumulation of ppGpp attenuates plant growth and development [23]. In conclusion, *AtYchF1* might be a critical regulatory factor in controlling the cytosolic ppGpp-mediated growth inhibition in plants (Table 2).

## 5. Conclusions and Outlook

YchF subfamily universally exists in both bacteria and eukarya except archaea [1,8]. The N-terminal G domain consists of five motifs that are highly conserved among all P-loop GTPases [14,50], of which G4 and G5 motifs determine specific ATPase or GTPase activities. YchF plays a critical role in regulating growth and stress responses among different organisms and life processes. In the current review, we have listed the important and conserved amino acid residues of YchF in not only Table 1 but also the interactive partner proteins in Table 2.

**Figure 4:**
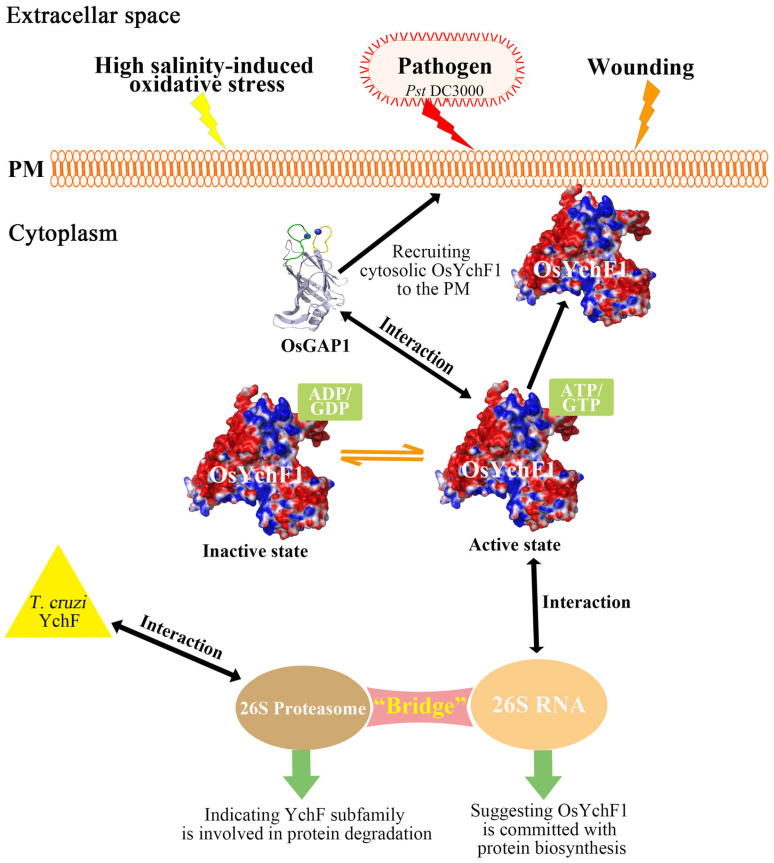
Working model to explain the structure-function relationship of YchF. The interaction of OsYchF1 (PDB code: 5EE0) and its activating protein OsGAP1 (PDB code: 4RJ9) participate in stress response. OsYchF1 is activated when binding ATP/GTP and inactivated when binding ADP/GDP. The possible role of members of the Ychf subfamily might be involved in protein balance. OsYchF1 can interact with OsGAP1 and 26S RNA, while *T. cruzi* Ychf can interact with 26S proteasome, suggesting that the Ychf subfamily may act as a bridge between protein synthesis and degradation.

The unique YchF is probably a guanosine or adenosine nucleotide-dependent translational factor associated with the ribosomal particles and subunits of the proteasome, potentially bridging the protein biosynthesis [1,24,51] and ubiquitin-dependent protein degradation to maintain proteostasis [26,32,33]. YchF is also involved in life response to environmental challenges by recruiting many partner proteins [1] (Figure 4). In prokaryotes, YchF might function as a GTP-dependent translation factor, participating in the translation process as part of the nucleoprotein complex [17,24,30,38] and being involved in oxidative stress response [18]. In yeast, as a representative of eukaryotes, a YchF homologous YBR025c is induced by H_2_O_2_ and participates in the degradation of damaged proteins by interacting with the 26S proteasome in response to oxygen stress [27]. The human Obg-like ATPase1 (hOLA1) is a human homolog of YchF that is overexpressed in several human malignancies and acts as a negative regulator of multiple oxidants [34,37].

OsYchF1 is a novel unconventional G protein in rice. The molecular mechanisms of *OsYchF1* in rice still remain largely unknown. The gain-of-function *OsYchF1* overexpression transgenic lines and loss-of-function *OsYchF1* knockout or knockdown mutants have not been constructed and monitored in rice, and the agronomic traits of *OsYchF1* need to be observed. Moreover, YchF is not only related to stress responses but also appears to influence the metabolic processes in diverse species, which should be delineated and clarified in the future.

## Figures and Tables

**Table 1:** Summary of function-related amino acid residues in YchF subfamily.

Homolog	Species	Residues	Location	Supportive Reasons/Effects	Functions	References
*E. coli* YchF	*Escherichia coli*	His114	A highly flexible loop of G domain	Supporting the flexible loop to reach a catalytically active conformation	Critical for ATPase activity (+)	[15]
*E. coli* YchF	*Escherichia coli*	Cys35	G2 motif	Allows YchF dimerization via a disulfide bridge	Critical for ATPase activity (−)	[16]
*E. coli* YchF	*Escherichia coli*	Lys78 (Arg)	G domain	YchF-K78A mutant shows similar hydrolysis activities in presence of Na^+^ or K^+^, but K78R mutant retained potassium specific stimulation of ATPase activity	Plays a key role in determining the potassium dependent ATPase activity	[17]
hOLA1	*Homo sapiens*	Leu96	G domain (next to G3 motif)	A conserved Gln residue involved in GTP hydrolysis in Ras-like GTPases has been replaced	Inactivates Ras-like GTPases	[14]
*E. coli* YchF	*Escherichia coli*	Ser16 (Ser36 in *H. sapiens*)	G1 motif	Ser16 phosphorylated when H_2_O_2_ absence; Dissociation of KatG	Supports the ATPase activity; Detoxifies H_2_O_2_	[14,18]
*E. coli* YchF	*Escherichia coli*	Leu76	G3 motif	Hallmark for HAS-NTPase	Slightly affects ATPase activity (+)	[15]
hOLA1	*Homo sapiens*	Thr37	G domain	The main chain amide of Thr37 contacts the α-phosphate of AMPPCP	Supports the ATPase activity	[14]
hOLA1	*Homo sapiens*	Ser36/Val33	G1 motif	The main chain amide of Ser36 and Val33 contacts the β-phosphate of AMPPCP	Supports the ATPase activity	[14]
hOLA1	*Homo sapiens*	Asn32	G1 motif	The main chain amide of Asn32 forms a hydrogen bond to the γ-phosphate of AMPPCP	Supports the ATPase activity	[14]
hOLA1	*Homo sapiens*	Asn230	G4 motif	Its mutation to alanine abolished nucleotide binding	Contribute to nucleotide binding	[14]
hOLA1	*Homo sapiens*	Leu231	G4 motif	Specificity for adenine binding is based on the interaction between the adenine N-6 group and Leu231 main chain CO in G4 motif	Make YchF preference for ATP rather than GTP	[14]
hOLA1	*Homo sapiens*	Ser310	TGS domain	The H-bond between Ser310 O_γ_ and the exocyclic N-6 of an adenine is formed in a position similar to the ppGpp O-6	Make YchF preference for ATP rather than GTP	[14]
hOLA1	*Homo sapiens*	Phe127	Coiled-coil domain	Mutating this residue to Ala diminishes ATP binding drastically	Contribute to base recognition	[14]
AtYchF1	*Arabidopsis thaliana*	Glu345	TGS domain	Conserved and solvent-exposed	Most critical for its interaction with the regulator, GAP1	[19]

Amino acids in brackets indicate that there are other known residues presenting at the same position in the orthologs; In the “functions” column, (+) means upregulation, and (−) means down-regulation.

**Table 2:** Summary of main cellular elements interacting with the members of the YchF subfamily.

Homolog	Organism	Interactive Factors	Effects	References
OsYchF1	*Oryza sativa*	OsGAP1	Activating OsYchF1 GTPase and ATPase activity	[2,19,21,22]
AtYchF1	*Arabidopsis thaliana*	AtGAP1	Activating AtYchF1 GTPase and ATPase activity	[2,19,21,22]
AtYchF1	*Arabidopsis thaliana*	ppGpp	*AtYchF1* might be a critical regulator in controlling the cytosolic ppGpp-mediated growth inhibition in plants	[23]
*E. coli* YchF	*Escherichia coli*	30S ribosome, 70S ribosome	The 70S ribosome act as an ATPase activating factor (AAF) to stimulate YchF’s ATPase activity	[17,24]
*E. coli* YchF	*Escherichia coli*	tRNA	YchF interacts with the 3′-CCA end of tRNA through its TGS-domain, indicating that YchF is involved in protein synthesis	[25]
*T. cruzi* YchF	*Trypanosoma cruzi*	26S Proteasome	*T. cruzi* YchF co-immunoprecipitates with a regulatory subunit of the *T. cruzi* proteasome, involving in protein degradation	[26,27]
*E. coli* YchF	*Escherichia coli*	KatG	YchF interacts with KatG and inhibit its catalase activity, revealing that YchF regulates the oxidative stress response	[16]
*S. cerevisiae* YchF	*Saccharomyces cerevisiae*	Eukaryotic translation elongation factor 1 (eEF1)	Supporting a role for YchF during translation	[28,29]
*E. coli* YchF	*Escherichia coli*	Translation initiation factor 3 (IF3)	YchF enhances the anti-association activity of IF3, stimulates the translation of leaderless mRNAs	[30]
*E. coli* YchF	*Escherichia coli*	Thioredoxin 1 (TrxA)	YchF dimer is dissociated by TrxA, which stimulates the ATPase activity	[16]
hOLA1	*Homo sapiens*	Eukaryotic elongation initiation factor 2 (eIF2)	hOLA1 effectively blocks the formation of TC (ternary complex) through its intrinsic GTPase activity, leading eIF2 unable to deliver Met-tRNA_i_^Met^ to the 40S ribosome to initiate translation	[31]
hOLA1	*Homo sapiens*	Heat shock protein 70 (HSP70)	OLA1 can interfere with the binding and function of the E3 ligase CHIP to HSP70, leading to the stabilization of HSP70, and response to heat shock	[32]
hOLA1	*Homo sapiens*	Superoxide dismutase 2 (SOD2)	OLA1 deficiency can enhance CHIP affinity for HSP70-SOD2 complexes, facilitating SOD2 degradation, supporting OLA1 plays a role in response to mitochondrial oxidative stress	[33]

## Data Availability

Not applicable.
